# A Discourse on Percutaneous Closure of the Patent Ductus Arteriosus in Premature Neonates: Controversy, Remedy, or Paradox of Choice?

**DOI:** 10.1016/j.cjcpc.2024.12.002

**Published:** 2024-12-24

**Authors:** Wisam Abozaid, Souvik Mitra, Bonny Jasani, Lee Benson, Steven Lee Rathgeber

**Affiliations:** a Division of Pediatric Cardiology, Department of Pediatrics, Faculty of Medicine, University of British Columbia, Vancouver, British Columbia, Canada; b Division of Neonatology, Department of Pediatrics, Faculty of Medicine, University of British Columbia, Vancouver, British Columbia, Canada; c Department of Neonatology, Hospital for Sick Children, Toronto, Ontario, Canada; d Division of Cardiology, Department of Pediatrics, the Labatt Family Heart Centre, the Hospital for Sick Children, University of Toronto School of Medicine, Toronto, Ontario, Canada

## Abstract

Whether conversations about the patent ductus arteriosus (PDA) are engaging and productive or controversial and inconclusive, they are commonplace within the neonatal intensive care unit. “To close or not to close” is often the final question that concludes these conversations. Even in the era when surgical closure was the only option for intervention, a consensus on the best management was typically difficult to achieve. The development and widespread adoption of percutaneous PDA closure in premature neonates has now added a new dimension to these conversations and converted the binary question to a more nuanced process that incorporates less invasive options. Because the procedure has been well established and shown to be safe in appropriately selected babies, it is timely to consider what, if any, aspects of the decision-making process have changed in the premature neonate. This article is a comprehensive review of the current literature on the minimally invasive transcatheter PDA closure procedure in premature babies and aims to examine the impact of this procedure on clinical decision-making in neonatology practice.

## Résumé

Les débats concernant la persistance du canal artériel (PCA), qu’ils soient stimulants et productifs ou controversés et peu concluants, sont chose courante à l’unité de soins intensifs néonataux. « Fermer ou ne pas fermer » est bien souvent l’ultime question qui conclut ces conversations. Même à l’époque où la fermeture chirurgicale était la seule option offerte, il était généralement difficile d’en arriver à un consensus quant à la meilleure façon de la prendre en charge. La mise au point et l’adoption généralisée de la fermeture percutanée de la PCA chez les nouveau-nés prématurés ont ajouté une nouvelle dimension à ces conversations et transformé une question binaire en un processus plus nuancé qui comprend des options moins invasives. Depuis que cette technique est largement utilisée et s’est révélée sans danger chez les nourrissons dûment choisis, il est opportun d’évaluer en quoi ces aspects du processus de prise de décision ont changé la donne pour les prématurés, le cas échéant. Cet article consiste en un examen exhaustif de la littérature médicale actuelle sur l’intervention minimalement invasive par cathéter visant à fermer la PCA chez les prématurés. Il vise également à examiner les répercussions de cette intervention sur le processus de prise de décision clinique en néonatologie.

The patent ductus arteriosus (PDA) has occupied the imaginations of cardiologists and cardiac surgeons for well over a century. Dr John Munroe, a surgeon in Boston, was the first to propose the benefits of surgical closure of the PDA and proposed (but did not perform) an operation for its ligation.^1^ Over 3 decades passed before the landmark PDA ligation performed by Dr Robert Gross in 1938 on a 7.5-year-old girl,^2^ laying the groundwork for ligation as a safe procedure and prompting further innovation in the field of congenital cardiology. In 1967, Porstmann et al.^3^ described a transcatheter technique for ductal closure using a polyvinyl alcohol foam material to occlude the duct. This option was reserved for young adults and older children, as the device was not suitable for young children due to the requirement for large arterial vascular access. Dr William Rashkind was the first to describe a transcatheter closure technique suitable for young children, which was first used in a 3.5 kg, 8-month-old infant in 1977.^4^ The prosthesis consisted of a single-disk grappling-hook stainless steel skeleton, filled with a cone of polyurethane/polyvinyl alcohol foam. Multiple implant and delivery system design changes were made until the double-disk device design was finalized and first used in 1981. Rashkind et al.^5^ reported their multicentre experience with transcatheter PDA closure between 1976 and 1986 in children as small as 6 kg (as young as 3 months), with promising results. Multiple PDA closure devices were subsequently introduced, and numerous studies demonstrated the safety, feasibility, and effectiveness of transcatheter PDA closure, including preterm infants.^6^, ^7^, ^8^, ^9^, ^10^, ^11^, ^12^ In 2003, the US Food and Drug Administration (US-FDA) approved the Amplatzer Ductal Occluder device (ADO I; Abbott Structural Heart, Plymouth, MN), a self-expanding implant made of nitinol wire for percutaneous ductal closure. However, the delivery system diameter limited its use to infants weighing more than 6 kg. A modification of the Amplatzer line of ductal occluders (ADO II AS), known as the Amplatzer Piccolo device, received CE-mark approval in Europe in 2011 for closing PDAs in patients weighing 6 kg or more and received the US-FDA approval for PDA closure in patients ≥700 g in January 2019. This device features a smaller design suitable for antegrade PDA closure in premature infants as small as 700 g.^13^

Although the simple patent arterial duct has inspired progress in the field of paediatric interventional cardiology, it continues to bewilder us as we engage in interdisciplinary conversations to decide whether its existence is consequential. In this review, we hope to explore the evidence, experience, and opinions surrounding PDA closure in premature infants that should be considered as we examine a prototypical conversation that could take place in any neonatal intensive care unit (NICU).

## Clinical Vignette

A neonatologist and second-year neonatology fellow are on clinical rounds with a 13-day-old infant born at 24 weeks and 2 days of gestational age (GA). The birth weight was 650 g, via spontaneous vaginal delivery to an otherwise healthy primigravida mother who presented with sudden onset preterm labour and preterm rupture of membranes. The baby was intubated at birth, received 2 doses of surfactant via the endotracheal tube due to respiratory distress syndrome, and received antibiotics due to suspected maternal chorioamnionitis. Despite 2 doses of surfactant, the baby continued to have high ventilatory and oxygen requirements (requiring a mean airway pressure of up to 12 cm H_2_O on high-frequency oscillatory ventilation and an FiO_2_ of up to 0.5 to maintain the SpO_2_ between 90% and 95%). A cardiology consult was sought on day 3 of life. The echocardiogram showed a structurally normal heart with a large, unrestricted duct shunting exclusively left to right, measuring 2.4 mm at the pulmonary end. There was evidence of left-sided volume overload, with a left atrium to aortic root (LA:Ao) ratio of 1.8. Evidence of systemic hypoperfusion was observed, characterized by reversal of diastolic flow in the descending aorta and the celiac trunk. According to the unit protocol, the infant was started on a 3-day course of intravenous ibuprofen. On day 5, the baby developed oliguria and hypotension, likely secondary to low diastolic blood pressures. An echocardiogram was repeated, revealing a large duct with significant dilatation of the LA and left ventricle. The clinical signs were primarily attributed to the duct; however, given the renal compromise, the medical team decided to initiate a 7-day course of intravenous acetaminophen. Over the course of the next 7 days, the oliguria resolved; however, the infant continued to require elevated ventilatory and oxygen requirements, with a mean airway pressure of up to 14 cm H_2_O and an FiO_2_ of 0.7 with persistent diastolic hypotension. A third echocardiogram obtained on day 13 demonstrated no major changes. It revealed a large, unrestricted 2.8-mm PDA shunting left to right, with high left ventricular (LV) output (420 mL/kg/min) and reversal of diastolic flow in the descending aorta.

Before clinical rounds, the neonatology fellow met with the parents, who were understandably very anxious due to their baby’s continued critical condition. This was partly due to the PDA, which persisted despite multiple unsuccessful attempts at medical closure. The following outlines the subsequent discussion between the neonatology fellow and the neonatologist.

### Neonatology fellow

The duct is still causing significant problems despite medical therapy, and the family is visibly stressed. I think we should definitively close this duct now.

### Neonatal staff

I agree that the duct is persistent, and you mentioned that it is causing “significant” problems. Could you clarify the criteria used to define a “significant duct”?

### Neonatology fellow

There is considerable debate about what we mean by a significant PDA. Despite over 138 clinical trials on PDA management that have enrolled nearly 12,000 preterm infants, we still lack a unified definition of what qualifies a haemodynamically significant PDA (hsPDA).^14^ However, a recent guideline on procedural closure of the PDA in preterm infants attempted to reach a consensus on this question through a rigorous e-Delphi survey involving 45 experts, including 13 general neonatologists and 32 neonatologists with expertise in haemodynamics.^15^ The consensus was that the definition of haemodynamic significance should be based on a combination of clinical signs and echocardiographic parameters.

With regards to clinical signs, *post hoc* analysis of the PDA-TOLERATE trial (n = 202; mean GA, 25.8 ± 1.1 weeks) showed that prolonged PDA exposure in infants requiring intubation for more than 10 days was associated with moderate to severe bronchopulmonary dysplasia (BPD).^16^ Similarly, a *post hoc* analysis of the TRIOCAPI trial^17^(n = 349; mean GA, approximately 26 weeks) demonstrated that the risk of BPD and death increased only when infants required intubation for at least 10 days (odds ratio [OR]: 2.41, 95% confidence interval [CI]: 1.47-3.95). Therefore, dependence on invasive mechanical ventilation for at least 10 days should be considered when selecting patients with PDAs that are candidates for closure.

### Neonatologist

I agree with your comment about persistent ventilation requirements. How do other clinical signs of poor systemic perfusion weigh into the decision?

### Neonatology fellow

That is a more challenging question. In terms of signs of ductal steal, there is currently no consensus on whether definitive shunt elimination should be considered in clinically stable preterm infants presenting solely with signs of reduced systemic perfusion, such as renal impairment or feeding intolerance, in the absence of clear signs of pulmonary over circulation as described earlier. However, you have not entirely stumped me, as the echocardiogram remains an effective tool for guiding evidence-based decisions when carefully integrated with clinical signs.

Several echocardiographic markers are used to assess the haemodynamic significance of a PDA. These markers are broadly categorized into measures of ductal size and flow characteristics (eg, direction and velocity of shunt flow), LV volume loading and pulmonary over circulation (eg, LV output, LA:Ao ratio, left pulmonary artery [LPA] diastolic velocity, and mitral valve E:A ratio), and markers of systemic hypoperfusion (eg, flow patterns in descending aorta, celiac trunk, or middle cerebral artery). A combination of these markers has been used in clinical trials and observational studies to define an hsPDA. A PDA diameter >1.5 mm and LA:Ao ratio >1.4 are the 2 most commonly used measures to define haemodynamic significance in randomized control trials (RCTs).^18^ However, significant variability in measuring these parameters can result in diagnostic imprecision.^19^ Regarding other echocardiographic findings, a prospective observational study of 75 infants (median GA 33 weeks) demonstrated that the echocardiographic assessment of reversed diastolic flow in the descending aorta had the strongest correlation with PDA shunt volume, as assessed by phase-contrast magnetic resonance imaging.^20^ In addition, a prospective observational study of 25 preterm infants (birth weight <1250 g) showed that a calculated LV output >300 mL/kg/min was associated with a symptomatic PDA.^21^ A few studies have attempted to define an hsPDA by combining multiple echocardiographic parameters. A retrospective study evaluated 242 infants ≤28 weeks of GA and showed that each week of exposure to an echocardiographically defined moderate (1.5-3 mm) or large (>3 mm) PDA (based on the staging criteria proposed by Sehgal et al^22^) was associated with an increased risk of chronic lung disease (CLD) (OR: 1.7, 95% CI: 1.09-2.66).^23^ It has also been demonstrated that earlier time to successful extubation in ventilator-dependent extremely preterm infants with large PDA diameters (>2.5 mm) and significant LV dilation (*z* scores >2) is associated with surgical ductal ligation. This suggests that these echocardiographic indices may accurately convey the severity of ductal shunting and its impact on pulmonary function beyond the first 2 weeks of life, which is typically when ligation is performed.^24^, ^25^, ^26^ A “PDA severity score” has been derived based on a prospective study that enrolled 141 infants born at GA <29 weeks and combined GA with echocardiographic characteristics at 24-48 hours after birth to provide an accurate prediction of the composite outcome of death or BPD (area under the curve: 0.92, 95% CI: 0.86-0.97).^24^^,^^25^ The score had a greater discriminatory ability than clinical indices alone and selected echocardiographic indices for inclusion based on a significant univariable association with the primary outcome. A recent before-and-after interventional study from Iowa showed that targeted PDA therapy using an echocardiographic PDA score >6, with early haemodynamic screening and physiology-guided care, reduced the primary outcome of death and/or severe intraventricular haemorrhage in preterm infants <27 weeks of gestation.^27^ The recently concluded SMART-PDA trial, conducted in Canada and the United States, exclusively enrolled infants born <26 weeks of GA and used criteria that categorized clinical and echocardiographic signs into mild, moderate, and severe. This provided clinicians with a treatment algorithm based on a combination of clinical criteria and echocardiographic measurements of PDA severity.^28^ Therefore, in consideration of the available evidence and the recent consensus statements on procedural PDA closure, it is conditionally recommended that definitive PDA shunt elimination may be considered if the infant shows signs of a large persistent left to right transductal shunt (PDA size >2.5 mm) in addition to one of (1) LV output >300 mL/kg/min and/or (2) holodiastolic flow reversal in descending aorta.^15^

Our neonate fulfils echocardiographic and clinical criteria for having an hsPDA as the infant has been dependent on mechanical ventilation for 13 days and has a 2.8-mm PDA with echocardiographic evidence of holodiastolic flow reversal in descending aorta.

### Neonatologist

You certainly know how to impress. You made a good case for the duct being haemodynamically significant. What if we decide to leave the duct untreated?

### Neonatology fellow

Are you sure you want to open that can of worms?! Well, there is evidence to support this competing viewpoint to leave the PDA untreated, which has led to vastly polarized opinions regarding treatment options. A recent cohort study of 39,096 preterm infants born at <28 weeks of GA across 139 NICUs demonstrated a wide variation in PDA treatment rates (13%-77% by NICU). Interestingly, both low and high PDA treatment rates were associated with increased risk of death or severe neurologic injury, suggesting that an ideal middle ground exists.^29^ Let me try to explain!

Observational studies have consistently demonstrated an association of prolonged PDA exposure in extremely preterm infants with adverse clinical outcomes, both in the short and long term. As previously mentioned, a cohort study of 242 preterm infants showed that each additional week of exposure to an hsPDA increased the risk to develop CLD (OR: 1.7), compared with infants with a small, nonsignificant PDA.^23^ A recently published Bayesian meta-analysis of observational studies demonstrated that prolonged exposure to a PDA shunt was associated with a higher risk of BPD-associated pulmonary hypertension.^30^ Furthermore, it has been shown that moving from proactive treatment to a conservative strategy in all infants born <1500 g resulted in a significant increase in BPD (34% vs 48%, *P* < 0.01) and a composite outcome of death or BPD (42% vs 57%, *P* < 0.01).^31^ In addition, a Canadian observational study comparing 2 sites in Montreal provided further evidence. One site changed their PDA management strategy to a nonintervention policy in 2013, whereas the other site continued with medical treatment and/or PDA ligation as indicated. This study demonstrated that the incidence of BPD/death increased by 31% in infants born <26 weeks of GA in the site that stopped PDA treatment, while the outcomes remained comparable in the 2 epochs at the site that continued active PDA treatment.^32^

### Neonatologist

Those are 2 very divergent approaches to management. I would assume that there must have been some justification for adopting an approach to not actively treat PDAs. Are you aware of any data that would support that approach?

### Neonatology fellow

There are a few considerations that may have led to this noninvasive approach. First, we must be cognizant that the PDA is a dynamic structure that can change spontaneously and is not always pathologic. In healthy preterm neonates >30 weeks of GA, the duct closes by day 4 in 90% and by day 7 in 98% of the cases, whereas spontaneous closure rates in infants <24 weeks of GA are only 8% and 13% by day 4 and day 7, respectively.^33^ Recent observational studies have further demonstrated that eventual spontaneous closure is achieved with conservative management, even in the smallest of infants. Semberova et al.^34^ showed that spontaneous closure occurs in the majority of extremely low GA (<26 weeks; 68%) and extremely low birth weight (ELBW) (<750 g; 76%) infants. Similarly, Sung et al.^35^ had shown that in extremely preterm infants (23-28 weeks of GA), 95% of infants with an hsPDA had a spontaneous PDA closure by discharge. In addition, data from recent large clinical trials on early PDA pharmacotherapy are enlightening when considering a less aggressive approach to PDA management, as these studies have failed to demonstrate a benefit for early attempts at medical closure of the PDA. For example, since 2020, 4 large RCTs (combined n = 1205) have been published on early ibuprofen therapy for PDA treatment in extremely preterm infants.^17^^,^^24^^,^^36^^,^^37^ These trials failed to demonstrate a difference in the outcome of BPD (relative risk: 1.07, 95% CI: 0.96-1.20), in keeping with the results of previous trials.^38^ From the aforementioned 4 trials, the Beneductus trial not only demonstrated noninferiority of an expectant management approach vs early pharmacotherapy, but in fact, the composite outcome of death/necrotizing enterocolitis/BPD was worse in the early treatment group (absolute risk difference: −17.6%, 95% CI: −30.2 to −5.0).^36^ With these recent trials combined, the mortality rate appears to be significantly higher in infants exposed to early ibuprofen than in those with no treatment (relative risk: 1.36, 95% CI: 1.03-1.80), which is indeed a cause for concern.^38^ However, it is unclear whether this reflects the elimination of an important role for the PDA in some patients or unanticipated harmful effects to the developing lung disease in those patients whose PDA was unresponsive to treatment.^28^

### Neonatologist

I can see how even a careful review of the data could result in a divided opinion on the ideal management of the PDA. Because you have clearly spent significant time reviewing the literature, what do you think might be contributing to the disparity in results between some of the observational studies and clinical trials?

### Neonatology fellow

I honestly found it difficult to synthesize the data and form a clear opinion, as it became evident from my review that there is a clear divergence between trial data and observational evidence. This highlights 2 major limitations of existing RCTs: (1) lack of representation of the most vulnerable preterm infants at the highest risk of PDA-attributable morbidity and (2) limitations in current definitions of hsPDA.

Most previous trials have included mature infants, with a mean GA of >26 weeks in 97% of trials.^39^ Interestingly, a follow-up analysis of eligible infants who were not enrolled in the recently published PDA-TOLERATE trial due to lack of physician equipoise showed that the group treated before 6 days of postnatal age had a significantly lower incidence of CLD and CLD/death despite having lower GA, less receipt of antenatal steroids, and substantially higher ventilatory requirements in the first few days of life.^40^ These findings suggest that exposure to a moderate-large PDA shunt for ≥1 week in an extremely preterm infant could lead to adverse clinical outcomes such as CLD or death irrespective of later PDA treatment.

The eligibility criteria with respect to the hsPDA definition have been broad, creating substantial heterogeneity in existing RCTs. As I highlighted before, a PDA size of >1.5 mm and an LA:Ao ratio of >1.4 have been the 2 most commonly used measures to define haemodynamic significance in RCTs.^18^ The major difficulty with this approach is that it does not differentiate between a “moderate” and “severe” PDA and completely ignores the clinical effects of the shunt volume. In fact, it has been demonstrated that PDA diameter itself is weakly correlated to shunt volume.^19^ Furthermore, a PDA of a particular size may have a variable haemodynamic effect based on the infant’s pulmonary mechanics. This could possibly explain why the Beneductus and the BABY-OSCAR trials, which enrolled preterm infants born <28 weeks of GA with a PDA with a left-to-right shunt of any diameter >1.5 mm, failed to demonstrate a benefit of early pharmacotherapy despite enrolling the smallest and sickest patients.^36^^,^^37^

### Neonatologist

Another important issue that we often overlook when we are interpreting these trials is that they are essentially assessments of “early provision of medical therapy,” and not specifically trials of “early elimination of the PDA shunt.” Given the poor response to medical therapy in a substantial proportion of infants, as evidenced by a high rate of open-label treatment, it is difficult to conclude whether the PDA is an unimportant problem or whether our available medical therapies fail to consistently achieve their intended effect in the smallest infants.^41^ Have you come across anything that may help reconcile some of the limitations of the current evidence?

### Neonatology fellow

To address your comments, trials investigating the clinical outcome following definitive PDA closure are ongoing. Unfortunately, only 109 babies have been randomized to trials of invasive PDA closure so far, which leaves us with very little trial evidence on whether definitive shunt elimination improves clinical outcomes.^14^ However, ongoing clinical trials such as the PIVOTAL (Preliminary Percutaneous Intervention Versus Observational Trial of Arterial Ductus in Low-weight Infants) trial being conducted across multiple centres in the United States randomizes extremely preterm infants with an hsPDA at 2-4 weeks of age to percutaneous transcatheter PDA closure vs conservative management. This trial should provide us with some definitive answers on whether definitive shunt elimination meaningfully improves clinical outcomes in the smallest babies.^42^ Expectedly, the current available evidence has resulted in a trend over time in Canada towards conservative PDA management without using cyclo-oxygenase inhibitors or procedural PDA closure, given the yet unproven benefit and potential harms of these treatments.^43^

### Neonatologist

That was an excellent summary of the existing evidence! That gives me a clearer picture on why evidence and practice fail to align when it comes to PDA management in preterm infants. Now that you have reviewed all the evidence, in your opinion, should we close it now, or should we wait?

### Neonatology fellow

Timing of definitive PDA closure is another topic of debate, as there are no definitive RCTs on the timing of shunt elimination. The only evidence that we have is circumstantial, derived from observational studies and *post hoc* analyses of RCTs; therefore, the overall certainty of evidence is low. A *post hoc* analysis of the PDA-TOLERATE trial showed that prolonged PDA exposure (≥10 days) was associated with moderate/severe BPD,^16^ whereas *post hoc* analysis of the TRIOCAPI trial demonstrated that the risk of BPD and BPD/death increased only when infants required intubation ≥10 days (OR: 2.41, 95% CI: 1.47-3.95).^44^ Further, a *post hoc* analysis of the PDA RCT in infants with a mean GA of 26 weeks showed that a persistent PDA beyond day 8 was associated with BPD/death (OR: 6.5 [1.7-25.5]).^45^ Based on such analyses, it appears that shunt exposure >8-10 days is associated with worse clinical outcomes. Conversely, an observational study of 224 preterm infants born at <28 weeks of GA showed that infants who underwent surgical PDA ligation before 10 days of age had an increased incidence of abnormal neurodevelopmental outcomes (OR: 11.44, 95% CI: 1.85-70.72; *P* = 0.01) compared with those with an open PDA who underwent ligation at ≥10 days or did not undergo ligation.^46^ Therefore, early PDA ligation (<10 days) may lead to worse neurodevelopmental outcomes than either delayed ligation (>10 days) or no ligation. On the basis of this evidence, our baby, who remains clinically unstable on mechanical ventilation with a large duct, is at a high risk for both short- and long-term complications, such as death, severe BPD, and BPD-associated pulmonary hypertension. Thus, I would suggest closing the PDA now.

### Neonatologist

You have made an excellent evidence-based argument to close the PDA without considering the potential complications from the procedure. Knowing that the neonate will be subject to procedural risk, how might this influence your approach?

### Neonatology fellow

On the basis of some reading that I’ve done, I think it is reasonable to consider percutaneous device closure. Should we discuss this case with the cardiology team to see whether this is a viable option?

### Neonatologist

You have introduced yet another element to add to our already spirited conversation. There is no consensus among neonatologists regarding the management of the PDA!^47^, ^48^, ^49^ Most of my experience has been with children undergoing surgical ligation, as the Amplatzer Piccolo device was only approved in 2019. Aside from reports of ducts being closed using off-label devices, the experience in our centre has been primarily with surgical ligation. I understand that our cardiology team is accepting referrals for neonatal PDA device closure, so this is worth considering for this child. Despite the emergence of the new percutaneous approach, surgical ligation remains an option and can be performed either through a thoracotomy or via a minimally invasive approach with video-assisted thoracoscopic surgery (VATS).^50^, ^51^, ^52^, ^53^ The less invasive modality of VATS for closure of PDA has been around since the early 1990s and is proven to be both feasible and safe. It has demonstrated good outcomes with a relatively short recovery period.^51^

### Neonatology fellow

I recognize that surgical ligation is a reasonable option and that the VATS approach is less invasive; however, I have been reviewing the recent experiences with percutaneous interventions and have read that in centres where device closure is an option, surgical ligation is typically reserved for neonates with unfavourable ductal morphology, active infection, or significant abdominal pathology.^54^ If our interventional cardiologists deem our patient a reasonable candidate for device closure, I believe that we should favour this approach and present the option to the family.

### Neonatologist

I am in favour of considering device closure for this child; however, we need to recognize that this would represent a deviation in our current centre’s practice, and we need more evidence to pursue a new intervention for our preemie population. I recently reviewed some literature that described a percutaneous intervention and surgical ligation as being equivalent with respect to outcomes and complications, whereas other reports have described a high rate of complications after a percutaneous intervention.^55^, ^56^, ^57^ I am also concerned about the potential for acute kidney injury (AKI) due to contrast exposure during a percutaneous intervention.

### Neonatology fellow

I accept that neither procedure is free of complications, and each has its own unique procedural and postprocedural risks. The primary complications associated with surgical ligation, whether by open thoracotomy or VATS, include postligation syndrome (PLS),^58^ with younger and smaller neonates being at higher risk of this complication;^59^^,^^60^ AKI;^61^ vocal cord paralysis;^62^^,^^63^ airway complications;^49^^,^^63^ bleeding; injury to thoracic duct resulting in chylothorax; pneumothorax; inadvertent ligation of the descending aorta, aortic arch, or LPA;^64^ and adrenal dysfunction.^52^ Surgical ligation, whether by open thoracotomy or VATS, is considered a safe and effective option while acknowledging the risk of these complications.^65^ A recent comparison between surgery and catheter-based closure, which included over 6000 babies, demonstrated a significant increase in the prevalence of device closure in very low birth weight neonates after 2018. The study showed that survival and complications were similar between the 2 groups, with a slightly lower incidence of complications after device closure in a subgroup controlled for a more recent era.^66^^,^^67^ Another meta-analysis also showed that transcatheter closure resulted in shorter hospital admission but had more residual shunts.^68^ In addition, a 2022 retrospective study using the Pediatric Health Information System database reported that postoperative stays were longer in premature infants who underwent surgical ligations compared with those who underwent trans-transcatheter closure.^69^ With respect to secondary AKI from the contrast load during device closure, modern iso-osmolar contrast agents are considered very safe in terms of renal toxicity. A retrospective review in children showed only 2 instances of AKI associated with contrast exposure in 2321 cases, where biomarkers of renal function were elevated after contrast exposure, without any clinically significant renal dysfunction.^70^^,^^71^ Another study specific to premature neonates included 160 cases, which showed no evidence of contrast nephropathy when compared with surgical ligation.^72^ In the Consensus guidelines for PDA closure using the Piccolo device in ELBW infants, for minimizing the potential of contrast-induced nephropathy, a minimal volume of contrast (2-4 mL) or no contrast in premature infants with pre-existing renal dysfunction was recommended.^13^ However, it is important to note that premature infants exposed to contrast are at a risk of developing hypothyroidism due to iodinated contrast exposure, and this should be kept in mind.^73^

### Neonatologist

You have certainly given this much thought, and you make a compelling case for the benefits of a percutaneous approach for this child. Perhaps we should discuss this further with the interventional cardiology team.

### Cardiologist

Good afternoon. I understand that you would like to discuss a neonate for possible PDA device closure.

### Neonatologist

Yes, thank you for meeting with us. With the help of our fellow, we have reviewed the data on the outcomes of percutaneous device closure and surgical ligation. We would like to get more information about the procedure to determine whether this is the right option for our little baby. To start, could you provide some details about the device or devices that can be used in this population?

### Cardiologist

**Figure 1:**
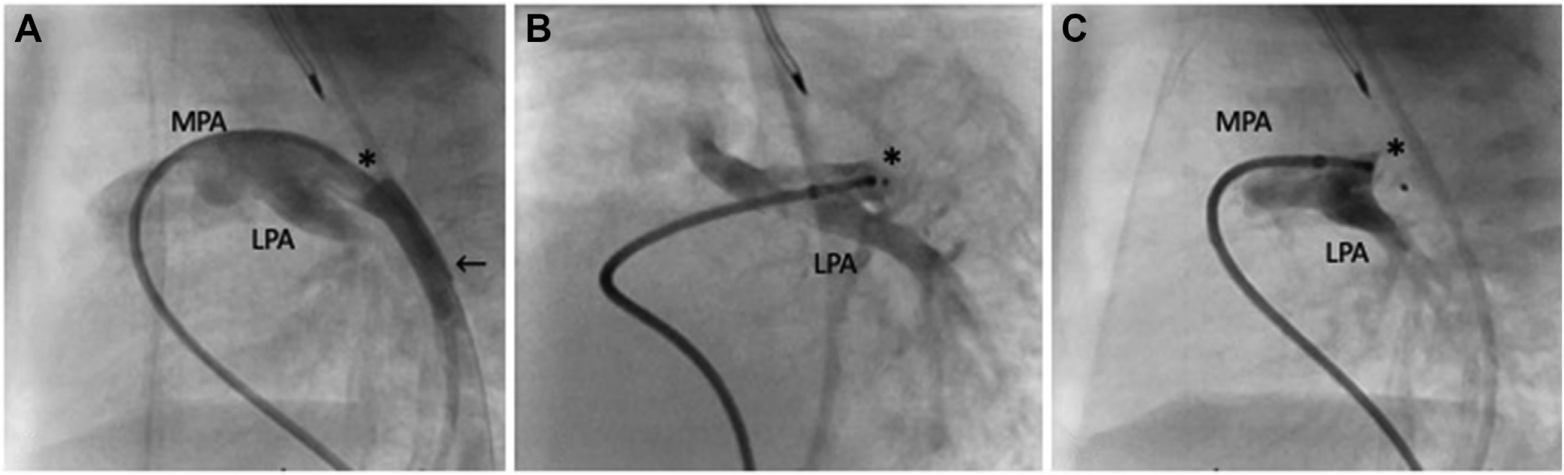
Lateral projection of a ductogram, duct (∗) and descending aorta (←) (**A**). Piccolo device in the duct (∗), still attached to the delivery cable. The left pulmonary artery (LPA) is unobstructed (**B**). Lateral projection of an injection through the delivery catheter while still attached to the implanted device (Piccolo, ∗) (**C**). MPA, main pulmonary artery.

I am happy to help! The Amplatzer Piccolo Occluder (Abbott Structural Heart) has received a lot of attention recently, as it was approved by the US-FDA for PDA closure in babies ≥700 g in January 2019.^13^ The device is a plug with 2 disks, one on each end. The plug portion comes in diameters between 3 and 5 mm; the disks are separated by 2-5 mm, with the disks 1.00-1.50 mm larger than the plug diameter (Fig. 1). The delivery system is 4 F in size, making it suitable for use in low birth weight and ELBW neonates.^74^ The early feasibility trial (for FDA approval) included 200 babies from multiple centres, half weighing 2 kg or less. The procedure was successful in 95.5% of babies and 99% in babies ≤2 kg in weight. Four babies (2.1%) had major complications (2 transfusions, 1 hemolysis, and 1 aortic obstruction), and there were 5 with acquired clinically significant tricuspid valve regurgitation due to trauma to the valve from the delivery catheter.^75^ Device embolization occurred in 5 babies, all successfully retrieved without surgical intervention. Survival at 3 years was 95.3% overall and 92.9% in children ≤2 kg. There were 9 deaths overall, none attributed to the procedure or the device. Another large study (645 babies) with device closure using the Piccolo device showed a success rate of 99.1%. There was a small incidence of device embolization (2%), LPA stenosis (0.4%), and aortic obstruction (0.5%). One procedure-related death occurred due to cardiac perforation.^76^

### Neonatologist

It seems like the outcomes are good in those who receive a Piccolo device, but are there limitations with respect to ductal size? If so, what are the options?

### Cardiologist

Yes, there are limitations with respect to PDA morphology, and suitable candidates can be screened based on images from a transthoracic echocardiogram. The Piccolo device is designed to close ducts ≤4 mm; therefore, there are a proportion of preemies who will not be suitable for this device. As such, there are other options available, although not specifically approved for PDA device occlusion. The Medtronic Microvascular Plug (MVP; Medtronic, Minneapolis, MN), an FDA- (December 2013) and CE-approved device for vascular embolization,^77^, ^78^, ^79^ has been successfully used in the preemie population and was used successfully before the application of the Piccolo device. It can be used to occlude ducts >4 mm, although there are some limitations due to the device’s length. It can be implanted using the same 4 F catheter used for the diagnostic portion of the procedure. The device has a nitinol framework covered partially by a polytetrafluoroethylene membrane. It has a football shape (*American*), with the uncovered end placed towards the aortic end of the duct, reducing the risk of arch obstruction. The device is available in several sizes, with the most commonly used being the MVP-3Q (for duct diameters 1.5-3 mm) and MVP-5Q (for duct diameters of 3-5 mm). MVP-7Q and MVP-9Q can close larger ducts 5-9 mm in diameters, but generally, this is beyond the range of a typical preemie’s patent duct.^80^ A multicentre experience with the MVP in premature infants included 15 neonates with a median weight and age at the time of the procedure of 1210 g and 4.5 weeks, respectively. Complete closure was observed in 93%, with 1 case of residual shunt that resolved during follow-up. No complications were reported.^80^ There is a single-centre study that describes closure of 22 large ducts >4 mm in diameter in neonates <2.5 kg using the MVP-7Q.^81^

The Amplatzer Vascular Plug II (Abbott Structural Heart) is another option that has been successfully used in premature neonates. It was initially developed for arterial embolization in the peripheral vasculature and received FDA approval in 2007.^82^ This device is a self-expanding nitinol cylindrical mesh with 3 equal-diameter disks, available in 3-, 4-, and 6-mm diameters. As this device elongates when it is constrained, there is the risk of LPA or aortic obstruction. The delivery cable is also stiffer than the other alternatives, which may not be well tolerated in some patients. Despite these challenges, the device has been successfully used in this population.^10^^,^^11^^,^^74^^,^^82^

### Neonatologist

Thank you for that excellent summary. I am glad to hear that there are a few options for devices that can be used safely in this population. What is the plan in the uncommon situation where the procedure is not successful?

### Cardiologist

Despite planning and preprocedure imaging, there are instances when we are unable to find an appropriate device to close a PDA either due to the morphology of the duct or because the duct is too large for the available devices. If this occurs, the alternative is to perform a surgical ligation. That said, there is evidence demonstrating that manipulation of the duct during an attempted PDA device closure has, in small cohorts, resulted in subsequent spontaneous closure.^83^ Due to this observation, it is prudent not to perform the ligation immediately but plan to re-evaluate the patient over the subsequent 24-48 hours to observe for ductal patency before scheduling a surgical procedure.

### Neonatologist

Thanks for elaborating on all the options. We have previously discussed the benefits of percutaneous closure relative to surgical ligation. What are the potential complications, both procedurally and in the postcatheterization period, that we will need to be aware of?

### Cardiologist

Although multiple studies have demonstrated reassuring outcomes and safety of device occlusion in preterm infants with low weights,^13^^,^^48^^,^^75^^,^^79^^,^^82^^,^^84^, ^85^, ^86^, ^87^, ^88^, ^89^ the procedure is not without risk. Haemodynamic instability, known as PLS, is not unique to surgical ligation and has been observed after device closure. It is characterized by decreased LV function, including decreased systemic blood pressure, LV dilation, and reduced ejection fraction, often requiring short-term administration of an inotropic agent (eg, milrinone). It has been reported in up to 23.3% of babies in a multicentre study, and in another study, it was observed in 15% of surgically ligated ducts vs none in the device occlusion group.^88^^,^^90^ In terms of evaluating patients at risk of PLS after device closure, a single-centre study showed that the risk of PLS, defined in that study as an ejection fraction of <55%, is higher in those with preprocedure LV dilation.^91^

Device protrusion into the LPA and descending aorta are additional procedural complications that can occur. Careful echocardiographic monitoring during the procedure can reduce or eliminate this occurrence. Any flow disturbance in the aorta caused by device protrusion into the aortic lumen often requires device repositioning or removal. There are reports describing LPA flow disturbances, noting that if the gradient is low, spontaneous improvement or resolution can occur, potentially avoiding the need for reintervention.^13^^,^^92^^,^^93^ In a study including 44 premature infants who underwent transcatheter closure, 1 child required LPA stent placement 3 months after the procedure. The remainder of the children who developed LPA (16%) or aortic (7%) obstruction experienced resolution over time.^92^ In another study, the incidence of LPA obstruction in ELBW infants undergoing device closure with the Piccolo device was 2%, whereas aortic obstruction occurred in 1.8%.^93^ These complications usually occur due to inappropriate device size (leading to protrusion into the aorta or compressing the LPA), difficulty in intraductal deployment, device movement after placement and release, or vasoconstriction of the pulmonary end of the duct, resulting in device migration.^13^^,^^75^

Device embolization is another complication of particular concern in ELBW infants, as surgical retrieval is very difficult. Device embolization has been reported in up to 8% in some studies and commonly occurs into a pulmonary artery branch, typically immediately after device deployment or within 24 hours of the procedure.^13^ Device embolization to the pulmonary artery in a baby >1 kg is usually well tolerated, with no reports of haemodynamic instability. However, in smaller babies, it can cause significant desaturation or signs of hypoperfusion if embolization occurs to the systemic circulation.^13^ If it occurs, the device can often be retrieved percutaneously, particularly if the embolization is detected at the time of the procedure.

Tricuspid valve regurgitation is another complication after device closure, with an incidence of 5% in babies ≤2 kg using the Piccolo device,^75^ primarily due to septal chord injury caused by the manipulation of the delivery catheter or long sheath.^13^ This complication can be minimized by reducing the number of wire and catheter exchanges and avoiding size discrepancies between catheter and wire combinations. Haemodynamically significant residual shunts and hemolysis are rare, accounting for 1% of the cases seen in the Piccolo feasibility trial,^75^ occurring mainly due to malposition or migration of the device. Postprocedure monitoring and assessment of device position during the first 24 hours after implantation are critical so that cases of device embolization may be identified and treated promptly.^13^ Vascular injury at the femoral vein access site and cardiac perforation have been reported but are extremely rare.^75^ The development of a technique for PDA device closure exclusively through femoral venous access was crucial for the success of PDA device closure in premature infants and eliminated the risk of arterial thrombosis. Given the small size of patients, routine surveillance of femoral vein patency is reasonable and practiced in some centres.

### Neonatologist

For future consideration, are there any absolute or relative contraindications that we should be aware of as we consider patients for device closure?

### Cardiologist

Contraindications include active infection, haemodynamic or respiratory instability that precludes transfer to the catheterization lab, intracardiac thrombus, severe renal dysfunction, and continuous right-to-left shunting across the PDA. PDA morphology does not generally preclude device closure, and suitability is more dependent on ductal diameter or length.^48^ High-frequency jet ventilation is not a contraindication to the procedure and has been described as being used during procedures without any compromise during device placement and without being associated with higher complication rates.^94^

### Neonatologist

Sometimes our patients are very fragile and do not tolerate handling and stimulation very well. Is there any experience doing device closure at the bedside instead of the catheterization lab?

### Cardiologist

Good question. It is not available at all centres and depends on the experience of the interventional cardiologist. There is a movement among interventionist to create an environment where device closure can be successfully performed in the NICU at the bedside, using adjunctive fluoroscopy or under echocardiographic guidance alone.^10^^,^^12^^,^^95^ The first example of using coils and Amplatzer duct devices in premature neonates with only echocardiographic guidance was described in 2011.^12^ The use of other devices with a combination of fluoroscopy and echocardiography has continued to be reported by others with good results.^10^^,^^86^^,^^96^, ^97^, ^98^ Early and midterm follow-up in a single-centre study showed good results, with 88% procedural success, 96% survival at the time of discharge, and no later deaths related to the procedure at 11 months of follow-up.^11^ A recent multicentre retrospective study, which included 53 percutaneous duct closures performed in the NICU, showed no procedure-related deaths. Four patients had major complications, including a pericardial effusion requiring pericardiocentesis, aortic coarctation requiring an aortic stent, device embolization, and 1 patient with late device migration causing severe right pulmonary artery obstruction.^99^

### Neonatologist

This has certainly been a productive conversation, and I have appreciated the evidence and experience that has been shared. Our fellow has presented an excellent argument that our patient would benefit from PDA closure at this time, and our cardiologist has enlightened me on the risks and benefits of percutaneous duct closure. If the duct is deemed appropriate for device closure after cardiology reviews the echocardiogram, we should refer the patient for closure.

## Clinical Vignette: Follow-up

The patient underwent a successful percutaneous transcatheter PDA closure in the cardiac catheterization lab 24 hours after referral in stable condition. The duct measured 2.5 mm at the PA end and was 8 mm long with a typical tubular morphology. A 4-2 Amplatzer Piccolo Occluder was successfully deployed under echocardiographic and fluoroscopic guidance. The patient was systemically heparinized and received 1 dose of intravenous antibiotics during the procedure. The patient was returned to the NICU in stable condition and remained ventilated for 72 hours before being able to be extubated to bilevel positive airway pressure. An echocardiogram that was performed 4 hours and 24 hours after catheterization demonstrated no residual shunt and no evidence of LPA or aortic obstruction. The LV function showed low-normal systolic function (ejection fraction: 53%) on the first postprocedure echocardiogram and normal systolic function 24 hours after the procedure. A femoral vein ultrasound 1 day after the procedure showed patent femoral veins with some narrowing of the right femoral vein but no obstruction. There was new mild tricuspid regurgitation that remained haemodynamically insignificant and resolved 1 month after catheterization. By that time the patient was stable on nasal continuous positive airway pressure and was following a typical trajectory for a premature infant.

This conversation illustrates both the substantial amount of data and experience that have accumulated in a relatively short period of time since the development and approval of first dedicated device to close ducts in premature babies in 2019, as well as the added nuance this has added to clinical decision-making. As for our query to start this conversation, neither “controversy” nor “remedy” accurately describes this procedure, as it is clearly widely accepted but does not supplant surgical ligation. Although there may be more substance to discuss whether the addition of percutaneous duct closure represents a “paradox of choice” alongside surgical ligation, the data suggest that these are not equivalent interventions. Percutaneous closure of the ductus in premature neonates represents an intervention with specific advantages, providing neonatal care more options to best manage these fragile patients.
